# The human voice areas: Spatial organization and inter-individual variability in temporal and extra-temporal cortices

**DOI:** 10.1016/j.neuroimage.2015.06.050

**Published:** 2015-10-01

**Authors:** Cyril R. Pernet, Phil McAleer, Marianne Latinus, Krzysztof J. Gorgolewski, Ian Charest, Patricia E.G. Bestelmeyer, Rebecca H. Watson, David Fleming, Frances Crabbe, Mitchell Valdes-Sosa, Pascal Belin

**Affiliations:** a Cente for Clinical Brain Sciences, Neuroimaging Sciences, The University of Edinburgh, United Kingdom; b Institute of Neuroscience and Psychology, University of Glasgow, United Kingdom; c Institut des Neurosciences de La Timone, UMR 7289, CNRS & Université Aix-Marseille, France; d Department of Psychology, Stanford University, Stanford, USA; e Cognition and Brain Sciences Unit, Medical Research Council, Cambridge, United Kingdom; f School of Psychology, Bangor University, United Kingdom; g Faculty of Psychology and Neuroscience, Maastricht University, The Netherlands; h Cuban Center for Neuroscience, Cuba; i Département de Psychologie, Université de Montréal, Canada

**Keywords:** Auditory cortex, Voice, Functional magnetic resonance imaging, Superior temporal sulcus, Superior temporal gyrus, Amygdala, Inferior prefrontal cortex

## Abstract

fMRI studies increasingly examine functions and properties of non-primary areas of human auditory cortex. However there is currently no standardized localization procedure to reliably identify specific areas across individuals such as the standard ‘localizers’ available in the visual domain. Here we present an fMRI ‘voice localizer’ scan allowing rapid and reliable localization of the voice-sensitive ‘temporal voice areas’ (TVA) of human auditory cortex. We describe results obtained using this standardized localizer scan in a large cohort of normal adult subjects. Most participants (94%) showed bilateral patches of significantly greater response to vocal than non-vocal sounds along the superior temporal sulcus/gyrus (STS/STG). Individual activation patterns, although reproducible, showed high inter-individual variability in precise anatomical location. Cluster analysis of individual peaks from the large cohort highlighted three bilateral clusters of voice-sensitivity, or “voice patches” along posterior (TVAp), mid (TVAm) and anterior (TVAa) STS/STG, respectively. A series of extra-temporal areas including bilateral inferior prefrontal cortex and amygdalae showed small, but reliable voice-sensitivity as part of a large-scale cerebral voice network. Stimuli for the voice localizer scan and probabilistic maps in MNI space are available for download.

## Highlights

• Three “voice patches” along human superior temporal gyrus/sulcus.
• Anatomical location reproducible within- but variable between-individuals.
• Extended voice processing network includes amygdala and prefrontal cortex.
• Stimulus material for “voice localizer” scan available for download.

## Introduction

An increasing number of functional magnetic resonance imaging (fMRI) studies confirm the existence in human auditory cortex of areas showing particular sensitivity to sounds of voice. These ‘temporal voice areas’ (TVAs) show greater response to voices, whether they carry speech or not, than to other categories of non-vocal sounds from the environment or to acoustical control stimuli such as scrambled voices and amplitude-modulated noise (Belin et al., 2000, Belin et al., 2002, Linden et al., 2011, Von Kriegstein and Giraud, 2004), and show a particular sensitivity to paralinguistic information (Bonte et al., 2013, Charest et al., 2013, Ethofer and Wiethoff, 2007, Ethofer et al., 2009, Ethofer et al., 2012, Formisano et al., 2008, Grandjean et al., 2005, Latinus et al., 2013, Moerel et al., 2012, Wiethoff et al., 2008).

The goal of the paper is (i) to provide a description of the TVA as observed using the localizer employed in all of our studies over the past 8 years, (ii) to examine inter-individual variability in the location of the TVAs, and (iii) to describe extra-temporal areas also showing voice-sensitivity. Although TVA activations observed in the brain of most adult individuals (but see Gervais et al., 2004) they are characterized by a large degree of variability in anatomical location. Group-level random-effects (RFX) maps of voice-selectivity suggest that the TVAs are organized in several clusters distributed antero-posteriorly along the superior temporal gyrus (STG) and sulcus (STS) bilaterally, but the precise anatomical location of the group-level peak varies noticeably across studies depending on stimuli and task (e.g. Belin et al. (2002): voice-sensitive regions − 62 − 14 0 and 63 − 13 − 1; Ethofer et al. (2012) emotional voice areas − 45 − 33 9 and 51 − 24 9; Grandjean et al. (2005) voice prosody − 60 − 24 0 and 62 − 30 6; Von Kriegstein and Giraud (2004) voice processing − 48 18 − 12/− 63 − 45 − 3 and 51 18 − 15/63 − 42 − 6; Warren et al. (2006) voice source − 62 − 18 − 2 and 62 − 26 0).

In contrast single-subject maps provide a somewhat different picture, with more clearly localized patches showing only a loose correspondence to peaks observed in group-level maps and a high variability across subjects. On observing this variability and the lack of obvious correspondence between single-subject, fixed-effect maps and group-level, random-effects maps, one is tempted to question the significance of peaks in random effects maps: is there any systematic structure in the location of the individual peaks of voice-selectivity, corresponding to e.g., several ‘voice patches’, perhaps with activations levels not consistent across subjects? Or are these peaks of voice-sensitivity an epiphenomenon without functional significance such as what would be expected in the case e.g., of a distributed code (Formisano et al., 2008). To address these issues we analyzed results obtained with the voice localizer in a large cohort of normal adult subjects. Specifically, we examined: (1) the overall “voice perception network” and intrinsic functional connectivity shown by the group-level RFX analysis of the contrast in the large group, and the involvement of extra-temporal areas; (2) the hemispheric lateralization of the voice-sensitive responses by performing a lateralization index analysis; (3) the inter-subject variability of this network by computing probability maps of the TVAs; (4) the variability of the peaks of voice-selectivity by performing a cluster analysis of individual voice-selectivity peaks; and (5) the reliability of the localizer by comparing results of the voice localizer ran twice in the same individuals.

## Methods

### Subjects

We scanned two hundred eighteen (*n* = 218; 117 males; age (mean ± SD) = 24.1 ± 7.0) healthy adult volunteers on the localizer scan as part of published and unpublished experiments of the Voice Neurocognition Laboratory (http://vnl.psy.gla.ac.uk/) of the Institute of Neuroscience and Psychology at University of Glasgow. Participants were recruited from the student population of Glasgow and were of various ethnic background, education and manual lateralization. Participants all provided written informed consent prior to participation, in accordance with the Declaration of Helsinki. The experiments were approved by the local ethics committee at University of Glasgow. During recruitment, subjects underwent a quick structured interview and filled a questionnaire about medical history, ensuring that all participants had no previous physical or mental health conditions.

### Localizer protocol and stimuli

The voice localizer consists of 10 min and 20 s block design with forty 8-s long blocks of either vocal (20 blocks) or non-vocal (20 blocks) sounds from stimuli already used in Belin et al. (2000). About 60% of the stimuli were recorded specifically for the localizer and the rest was taken from public databases available in year 2000 as well as from recordings of American English vowels from (Hillenbrand et al., 1995). For the current experiments, stimuli were presented using Media Control Functions (DigiVox, Montreal, Canada) via electrostatic headphones (NordicNeuroLab, Norway; or Sensimetrics, USA) at a comfortable level (80–85 dB SPL).

**Fig. 1:**
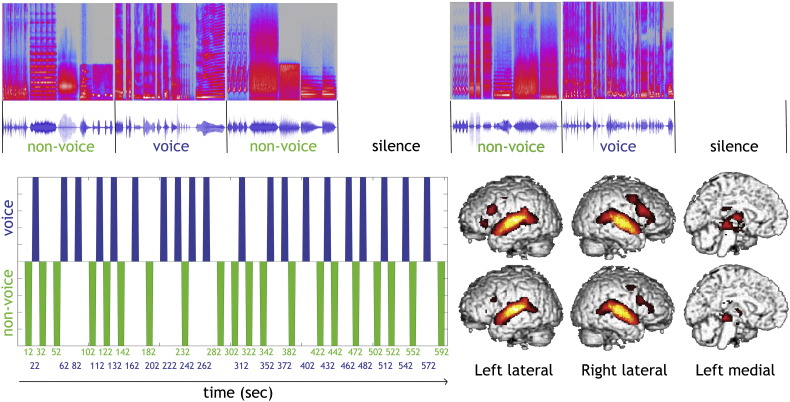
Voice localizer design. At the top is shown the first 7 blocks of the design with spectrograms (upper part, *x*-axis: time; *y*-axis: frequency, 0–11.025 kHz) and waveforms (lower part) of the 8-s blocks of non-vocal, vocal and silence periods. Block onset starts 2 s after experiment onset to allow a 2 s scanning period during which no stimulation is presented for sparse sampling designs (TR = 10s); however the design is also suitable for continuous scanning (TR = 2 s). At the bottom is shown the full design in time (voice in blue and non-voice in green) with the (non-convolved) blocks indicated. On the right hand side is shown the random effect (FWE 5%) for voice and non-voice stimuli separately that are then contrasted to reveal the TVA.

The 40 blocks are intermixed with 20 periods of silence allowing the hemodynamic to relax (Fig. 1). The relative power spectra of voice and non-voice blocks after convolution are similar, avoiding bias due to data sampling. Blocks are made of a mixture of either vocal sounds or non-vocal sounds, with at most a 400 ms delay between consecutive stimuli. The order of stimuli was attributed randomly, but is fixed for all subjects. Vocal blocks contain only sounds of human vocal origin (excluding sounds without vocal fold vibration such as whistling or whispering) obtained from 47 speakers (7 babies, 12 adults, 23 children and 5 elderly people) and consist of speech sounds (words, syllables or sentence extracts — 1 in English, 1 in French, 3 in Finnish, 2 in Arabic) and non-speech sounds (emotional positive or negative sounds like laughs, sighs, or cries, and neutral sounds like coughs, and onomatopoeias). The median ratio speech, non-speech is of 22.5% (min 0% max 75%). Non-vocal blocks consist of natural sounds (from natural sources like falls, sea waves, wind, and from various animals like cats, dogs, lions, and elephants) and of man-made sources (from objects like cars, glass, alarms, and clocks, and from (classical) musical pieces). All source categories are easily recognizable; although specific exemplars might have not been known to all participants (e.g. we can recognize an animal sound without identifying the species). Details about sound number, durations, amplitude and frequency can be found in Table 1. Stimuli (16 bit, mono, 22,050 Hz sampling rate) are normalized for RMS (same normalization for all stimuli); a 1-kHz tone of similar energy is provided for calibration. Stimuli are available for download at: http://vnl.psy.gla.ac.uk/resources.php.

### fMRI scanning

All scans were acquired on a 3 T Siemens (Erlangen, Germany) Tim Trio scanner at the Centre for Cognitive Neuroimaging (http://www.ccni.gla.ac.uk/), University of Glasgow. All fMRI data were acquired using a single-shot gradient-echo echo-planar imaging (EPI) sequence with the following parameters: field of view (FOV) = 210 × 210 mm^2^, 32 slices per volume, interleaved slices order, voxel size 3 × 3 × 3.3 mm^3^, acquisition matrix 70 × 70, flip angle = 77°, echo time (TE) = 30 ms. The repetition time (TR) was 2 s with an acquisition time (TA) of 2 s resulting in quasi-continuous scanning noise. In total 310 EPI volumes were acquired. In addition to the EPI data, a high-resolution 3D T1-weighted sagittal scan was acquired for each subject (voxel size 1 mm^3^ isotropic; acquisition matrix 256 × 256 × 192). Subjects were scanned while passively listening to the stimuli and keeping their eyes closed.

### fMRI data analysis

#### Pre-processing

MRI data were analyzed using SPM12b (r6080 — Wellcome Department of Cognitive Neurology, University College London). Pre-processing of functional scans consisted of slice timing (sinc interpolation — reference slice 31, i.e. middle of the TR — Sladky et al., 2011), motion correction (6 parameter affine transformation, realignment to the mean image, mean created using 4th spline interpolation), co-registration of the T1 image to the mean EPI (normalized mutual information), and of these images to the SPM average 152 T1 image (normalized mutual information — this step allowed the zero coordinate to be as in the template space). Finally, functional (3 mm isotropic voxels) data were normalized to the Montreal Neurological Institute (MNI) space (segmentation, and diffeomorphic normalization using the forward deformation field computed during segmentation, data resampled at 2 mm isotropic with a 4th degree B-spline interpolation, i.e. the midway between the functional (3 mm) and the structural (1 mm) image resolutions, — Ashburner, 2007) and spatially smoothed (Gaussian kernel of 6 mm full width at half maximum).

#### Random-effect analyses

First-level (single-subject) analysis used a design matrix containing separate regressors for vocal and non-vocal sounds, plus realignment parameters to account for residual motion artifacts as well as outlier regressors (Siegel et al., 2014). These regressors corresponded both to scans with large mean displacement and/or weaker or stronger globals. Outliers were defined using a modified boxplot rule (Carling, 2000). Vocal and non-vocal regressors were obtained by convolving boxcar functions representing the onset and offset of stimulation blocks by the canonical hemodynamic response function (HRF). The design matrix also included a filter at 128 s, and auto-correlation was modeled using an auto-regressive matrix of order 1. Once the model estimated, a contrast image vocal > non-vocal was computed. At the 2nd level, the contrast images from each participant were entered in a one-sample *t*-test, expressing at each voxel the likelihood that vocal > non-vocal contrast values are significantly different from 0 across the group. Significant voxels were examined at *p* = 0.05 FWE corrected (based on Gaussian random field — Worsley and Friston, 1995), and labeling was performed using the Anatomy toolbox (Eickhoff et al., 2005). Effect sizes are reported as percentage signal change (PSC), computing for each subject PSC maps with a scaling factor of 0.0132 (Pernet, 2014), then extracting the 1st eigen value across voxels for each region of interest (ROI) considered and finally averaging across subjects. The ROIs correspond to areas found in the random effect analysis with a further segregation of the TVA into 3 sub-regions, as obtained by the cluster analysis. For each ROI, 95% confidence interval of the mean PSC was obtained using a percentile bootstrap (Wilcox, 2012).

In order to investigate further what was driving the response in the different voice-sensitive regions, an event-related design analysis was performed. Voice blocks were split into 3 types of events: emotional stimuli, neutral stimuli, and speech. Non-voice blocks were split into 4 types of events: animal stimuli, natural stimuli, music and sounds derived from man-made objects. It is important to note that the voice localized was not designed to investigate the response to these various categories and it is therefore not optimal in terms of stimulus dispersion or sampling. This analysis provides however some insight on which categories elicited stronger responses. As before, the design matrix at the subject level contained separate regressors for vocal (x3) and non-vocal (x4) sounds, plus realignment parameters and outlier regressors. Effect sizes were computed as for the block design analysis, i.e. transforming the beta parameter maps into PSC (scaling factor of 0.0132) and then computing the 1st eigen value across voxels for each ROI. Percentile bootstrap on mean differences between vocal stimuli (emotional vs. neural, emotional vs. speech, neutral vs. speech) and between non-vocal stimuli (animal vs. man-made, animal vs. natural, animal vs. music, man-made vs. natural, man-made vs. music, natural vs. music) were computed to test if some categories were driving the BOLD response. For each ROI, the type one error rate was controlled using the Hochberg's step-up procedure but no control over the 18 ROIs considered was performed, the goal being to establish the response profile per ROI. Once the model estimated, a contrast vocal > non-vocal was obtained per subject and compared to the standard block analysis using a paired *t*-test. Similarly, differences between categories were computed separately and tested at the group level using a one-sample *t*-tests (family-wise error corrected *p* < 0.05).

#### Connectivity analysis

Functional connectivity analysis was performed between the areas observed at the group-level analysis. From the block design GLM, residuals were obtained and Pearson's correlations were computed between the eigen time-series of 18 regions of interest (ROIs): TVA anterior, mid, posterior (defined from form the cluster-analysis, see below), the IFG ventral and medial, the precentral gyrus, and subcortical structures. Eigen time-series are the eigen value across ROI voxels for each image acquired in time. The resulting connectivity matrices were z-transformed and the connectivity profile tested at the group-level using a topological permutation *t*-test, to ensure a strict control of the type 1 family-wise error rate.

First, connectivity matrices of each subject are permuted randomly and *t*-tests are computed for each connection (z-transformed correlations). Second, the maximum *t*-value across connections is stored. The procedure is repeated 10,000 times, giving a distribution of maximum *t*-values for the whole connectivity matrix. Third, the observed connectivity matrix is thresholded for *t*-values bigger than the 95th percentile of the permutation based distribution of maximum *t*-values.

#### Lateralization analysis

Hemispheric lateralization of the TVA was assessed using the LI-tool (Wilke and Lidzba, 2007). For each subject, a lateralization index (LI) was computed (i) over the whole brain (excluding 10 mm around midline), (ii) in the TVA mask defined by the random effect analysis and (iii) for the temporal lobes, based on bootstrapped LI curves (Wilke and Schmithorst, 2006) [15]. Following Wilke and Schmithorst (2006), only LI values above 0.2 or below − 0.2 were considered as reliable evidence for lateralization. In addition, we also estimated the lateralization by computing the average LI along with their 95% percentile bootstrap confidence intervals (Wilcox, 2012). *P*-values were obtained by considering the number of bootstrapped data (under H1) with values below − 0.2.

#### Probability maps

To evaluate the inter-subject variability of the TVA (robust versus variable signal within and between subjects), we computed a probability map based on individual thresholded data. For each subject, the t-map vocal > non-vocal was thresholded at *q* = .05 corrected (correction for multiple comparisons based on spatial extent (Chumbley et al., 2010)). However, rather than using a default cluster forming threshold for all subjects, this threshold was set individually using a Gamma–Gaussian mixture model. The voxel T-distribution is fitted with 3 different non-central models (Gaussian only, positive gamma and Gaussian, positive and negative gamma plus Gaussian) effectively separating the null voxel distribution from the ‘active’ voxel distribution (Gorgolewski et al., 2012). This method is more sensitive (less false negative) and allows a better delineation of the cluster spatial extent. Once the 218 thresholded maps were obtained, they were binarized, summed and normalized to 100 to create a probability map.

#### Clustering analysis

There is a trend towards describing functional regions (and specially localizers) on the cortical surface coordinates instead of in the volume. Frost and Goebel (2012) show that curvature driven cortex based alignment removes a substantial portion of macro-anatomical variability across subjects, yet variability in the observed spatial location of functional localizer regions remains which probably reflects the “true” functional variability. Therefore here we also describe the probabilistic location of TVA cluster centroids using a surface-based approach.

Cluster analysis was performed on the location of individual peaks found in the t-maps for the vocal > non-vocal contrast across a group of 218 subjects, in order to determine if they could be grouped into distinct clusters. Analysis was performed using the following steps for each individual subject: (1) Pial and white/gray matter surfaces were extracted with freesurfer (http://surfer.nmr.mgh.harvard.edu/) (Dale et al., 1999), subsampled to 81,924 vertices and all subjects brought into anatomical correspondence by spherical co-registration to the average template (Fischl et al., 1999). Then the midgray surface was calculated by averaging the coordinates from corresponding vertices of the pial and white/gray matter surfaces; (3) the anatomical T1 was then co-registered with the mean functional EPI image, and the co-registration transformation parameters were used to warp the midgray surface into the fMRI native space; (4) the unsmoothed fMRI time series (after slice timing and, movement correction in SPM8) were sampled (with spatial interpolation) on all surface vertices; (5) the time series on the surface were high pass filtered with a 128 s time constant; (6) a GLM using the localizer design matrix (vocal and non-vocal regressors plus realignment parameters and the session constant term) was used to obtain betas on the surface. The contrast vocal > non-vocal was calculated at each vertex; (7) a threshold was obtained for these individual t-maps using a false discovery rate with *q* = 0.05 and the default cluster forming threshold *p* = 0.001 (Chumbley et al., 2010); (8) the 10 largest peaks in each individual were identified in each hemisphere; this choice in the number of peak was arbitrary, but sufficiently large to reveal clustering of activity; (9) disks of 3 mm were created around each peak using functions from the Fast marching Toolbox (http://www.mathworks.com/matlabcentral/fileexchange/file_infos/6110-toolbox-fast-marching) and the Surfing Toolbox (http://sourceforge.net/projects/surfing/) and a weight of 1; (10) the 10 disks were summed, resulting in a density map for each subject; (11) a permutation procedure was used in which the peaks were then randomly positioned within the surface (excluding the medial wall) 1000 times, and the max of the density was measured. The histogram of this max over all the permutations was used to determine the *p* < 0.01 threshold; (12) connected clusters in the thresholded density map were found with the clustermeshmap.m function from the Neuroelf toolbox (http://neuroelf.net/). We report here the maxima for each cluster.

#### Analysis of test–retest reliability

Ten subjects were scanned twice with a delay ranging from 1 to 27 days between scans. Intra-class correlation coefficients (ICC) have been computed for each voxel of the brain using the third class of ICC as defined by (Shrout and Fleiss, 1979)

(1) $$ICC=\frac{MSS-MSE}{MSS+\left(k-1\right)MSE}$$

with *MSS* the mean square related to subjects and *MSE* the mean square error from a repeated measure ANOVA, and *k* is the number of scanning sessions. ICC can be viewed as a type of correlation. However, because it operates on data structured by groups rather than data structured as pairs, ICC is better seen as a measure of discrimination between subjects (Bland and Altman, 1996). Theoretically, ICC describes how strongly observations from the same subjects resemble each other with value ranging from 1 (the signal amplitude is identical from session to session) to 0. Using the ANOVA model, negative ICC can be obtained but are of no interest, simply indicating that the MSE is too large.

## Results

### Single-subject analysis

**Fig. 2:**
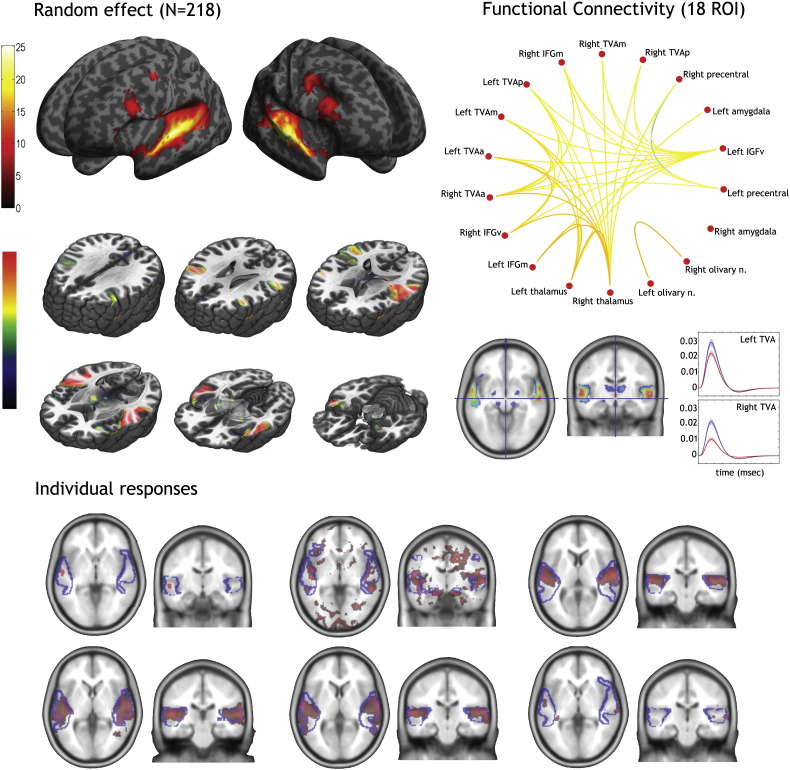
Random effects analysis in 218 individuals. At the top is shown the whole-brain voxels at which a *t*-test of the vocal vs. non-vocal difference in BOLD parameter estimates across the *n* = 218 subjects yields significant values (*p* < 0.05, FWE corrected) and the corresponding functional connectivity over the 18 ROIs selected. Data are projected (i) on the inflated cortical mesh surfaces created using Freesurfer version 4.0.1 from an average of 27 T1 scans of the same subject, and available within SPM, and (ii) on slices of the 152 EPI templates. Note the large undifferentiated cluster of significant voxels in the temporal lobes without clear maxima, and the involvement of many extra-temporal structures. At the bottom are shown data from 6 subjects (3 atypical and 3 typical) with the TVA from the RFX outlined.

Single-subject adaptive thresholding showed bilateral activations in ~ 94% of subjects (205 subjects out of 218). Three subjects did not show significant activations, although for 2 of them the signal was stronger for voice than non-voice stimuli, and 10 subjects showed unilateral activations (5 left-sided and 5 right-sided). Individual activation foci were fairly variable in significance level and extent yet consistently occurred along mid-STS bilaterally. Fig. 2 illustrates this variability in six individuals.

### Group-level analysis

The second-level random effects analysis offered a markedly different picture (Fig. 2 and Table 2). As for individual subjects, highly significant effects along extended portions of the temporal lobes (including areas TI1–TE10–TE11–TE12) were observed. The high statistical power associated with the large number of subjects in this group-level analysis highlighted however a number of extra-temporal areas not usually observed on single-subject contrast images, or even smaller group sizes. In particular there was clear involvement of regions of the prefrontal cortex, including in the upper part of the precentral gyri and of two clusters in the inferior frontal gyrus (IFG) in the ventral and medial part, laterally to Broca area (BA44/45). These IFG clusters of prefrontal voice sensitivity were located bilaterally in nearly symmetrical locations, but with higher significance and cluster size in the right hemisphere. The RFX analysis also highlighted subcortical structures: the superior olivary nuclei, the thalamus in both the temporal and prefrontal connected regions, and the amygdalae (SF).

Comparison of the block vs. event models showed no significant differences, suggesting that events can be used to estimate categorical responses. Investigation of the differences between a-priori categories revealed that in all areas but the thalamus, voice stimulus categories show the same pattern of activation with the lowest activation for emotional stimuli, then neutral stimuli and the highest activation for speech stimuli (Supplementary Table 1). In the left/right TVA emotional stimuli activated significantly less than neutral stimuli and speech, but neutral stimuli and speech did not differ significantly. The same pattern of results was observed in frontal regions, but the right ventral IFG. In that region, emotional and neutral stimuli did not differ and only speech stimuli showed stronger activations. No differences were observed in left/right amygdala and left/right thalamus between the three categories of vocalizations. In left olivary nucleus, emotional stimuli showed a significantly lower activity than speech stimuli. In right olivary nucleus, emotional stimuli showed a significantly lower activity than neutral stimuli. Similar analysis on non-voice stimuli showed much more variable results across ROIs (Supplementary Table 2), with music showing the highest activations except in the olivary and thalamic nuclei where animal calls showed higher activations. Overall, man-made object sounds and natural sounds elicited little activation compared to animals and music such as in the TVA, they show significantly less activation than animal calls and music. Whole-brain pair-wise analyses between categories showed that speech and neutral voices elicited stronger TVA activations than any other non-vocal categories. Emotional stimuli however, only showed stronger activations of the TVA compared to natural sounds and man-made object sounds. For music, stronger activations were observed at the temporo-occipital junction, posterior to the TVA. No differences were observed when compared to animal calls (Supplementary Fig. 1).

### Connectivity analyses

Functional connectivity was computed over 18 (bilateral) ROIs: 3 bilateral regions over the temporal cortex (see below cluster analysis results) and the 3 frontal and 3 subcortical regions observed in the random-effect analysis. Results showed positive correlations between all of these regions, with most regions showing many significant connections (Fig. 2). Of special interest here are the connections of TVA. All left and right TVA anterior/mid/posterior regions were connected with other ROIs except the left/right amygdala and left/right olivary nuclei, and the left thalamus. Amygdalae and olivary nuclei were interconnected and also connected to the left/right thalamus, which connected also to all frontal regions. This pattern therefore suggests continuous interaction (feed-forward and feed-back) between the TVA with other frontal voice-sensitive areas, with effect of subcortical structures via thalamic connections.

### Lateralization

Results show no evidence for a significant right hemispheric lateralization at the group level. The group effect map showed for the whole-brain analysis, an LI of 0.01 (mean 0.017, weighted mean 0.012), while the mean voxel count across subjects shows an LI of − 0.09 [− 0.12 − 0.05]. When restricting the analysis to all voice-sensitive regions, very similar results were obtained (mean 0.015, weighted mean 0.011, mean voxel count − 0.04 [− 0.07 − 0.003]) indicating that the RFX thresholded map reflects the true effect, independently of the statistical threshold used. Finally, when restricting the analysis to the temporal lobes, the RFX map showed a mean LI of 0.044 and weighted mean of 0.024, but a mean voxel count of − 0.08 [− 0.11 − 0.04].

At the individual subject level, 11% (whole brain), 19% (voice areas) and 13% (temporal lobe) of subjects showed a left lateralization (lower bound of the bootstrap CI > 0.2) and 33% (whole brain), 24% (voice areas) and 33% (temporal lobe) of subjects showed a right lateralization (upper bound of the bootstrap CI < − 0.2).

### Probability maps

**Fig. 3:**
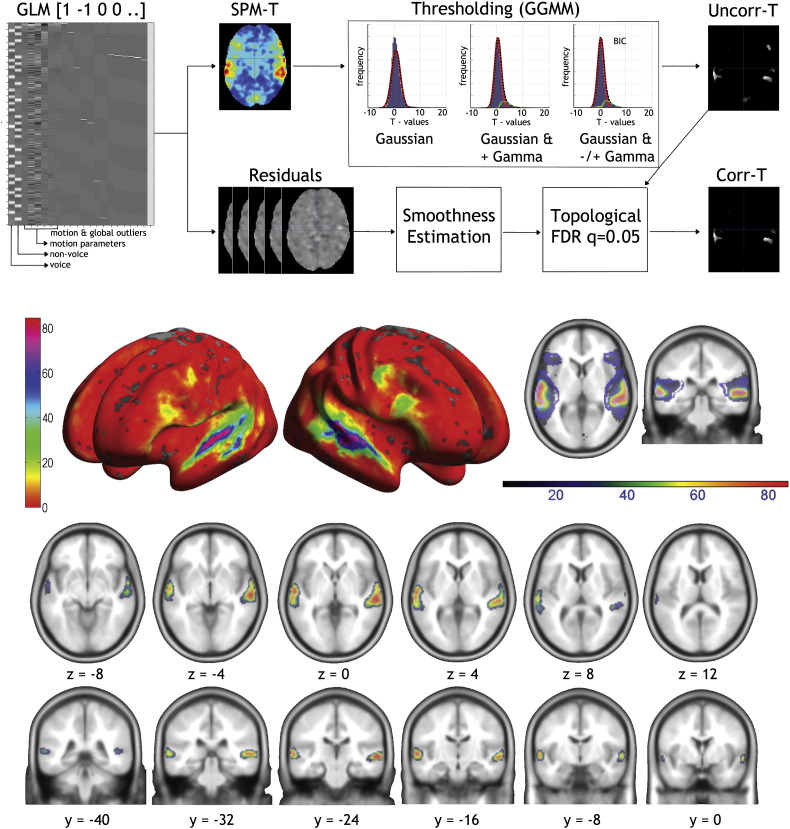
Probability maps of the TVA. At the top is illustrated the thresholding procedure: starting from the GLM output the SPM-T map is thresholded using a Gaussian–Gamma mixture model (the best model is selected using the Bayesian Information Criteria; BIC) yielding a thresholded uncorrected map (Uncorr-T). This map is then corrected for multiple comparisons using topological FDR, as implemented in SPM, yielding to a corrected map (corr-T). The average of all these maps across subjects forms the probability map shown below. The middle and bottom panels show the probability of activations at the individual level. Data are projected (i) on the inflated cortical mesh surfaces created using Freesurfer version 4.0.1 from an average of 27 T1 scans of the same subject, and available within SPM, and (ii) on slices of the 152 EPI templates.

Despite ~ 94% of subjects showing individual activations using the Gaussian–Gamma mixture model thresholding (Fig. 3), the maxima of the probability map were around 85%, which indicates a large inter-subject variability in individual maxima. The mean *t*-value threshold to create the individual maps was at 2.4 [2.3 2.5] and the average cluster size (corresponding to the left or right TVA) was 1412 [1177 1731] voxels. The maximum subject overlap (85.78% = 187 out of 218 subjects) was observed over the left (MNI [− 60 − 14 0]) and right (MNI [60 − 26 0]) STS. In frontal areas, only 15 to 20% overlap was observed and very few subjects showed significant activations in subcortical areas (overlap = 1 to 3%). In total, 78.9% of subjects overlapped in both hemispheres. Fig. 3 shows that thresholding the probability maps at 50% (dark/light green boundary) reveals largely symmetrical zones of common activation along the left/right STG in the posterior–anterior direction, more medially over the posterior part of the right STG.

### Cluster analysis

**Fig. 4:**
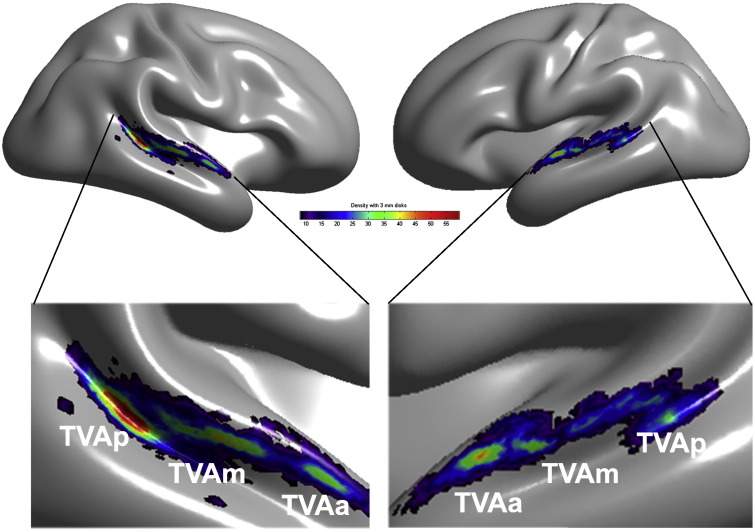
Cluster analysis. Shown on a 3D template of the gray/white interface is the density map representing the number of individual voice > non-voice activation peaks within 3-mm disks on the cortical surface (cf. Methods). The density map reveals three main clusters of voice sensitivity in each hemisphere along a voice-sensitive zone of cortex extending from posterior STS to mid-STS/STG to anterior STG. The cluster with the greatest peak density is in right pSTS, consistent with individual images (cf. Fig. 2).

The surface-based analysis of density of local activation peaks highlights a bilateral zone of voice-sensitivity extending from posterior STS to anterior STG bilaterally (Fig. 4) with three clear clusters in bilateral pSTS, in mid-STS/STG and anterior STG. The maximum peak density is observed in right pSTS, close to the fundus of the sulcus where STS is widest, consistent with the probability maps of Fig. 4. Table 3 indicates MNI coordinates of the three cluster centers in the left and right hemispheres.

### Test–retest reliability

**Fig. 5:**
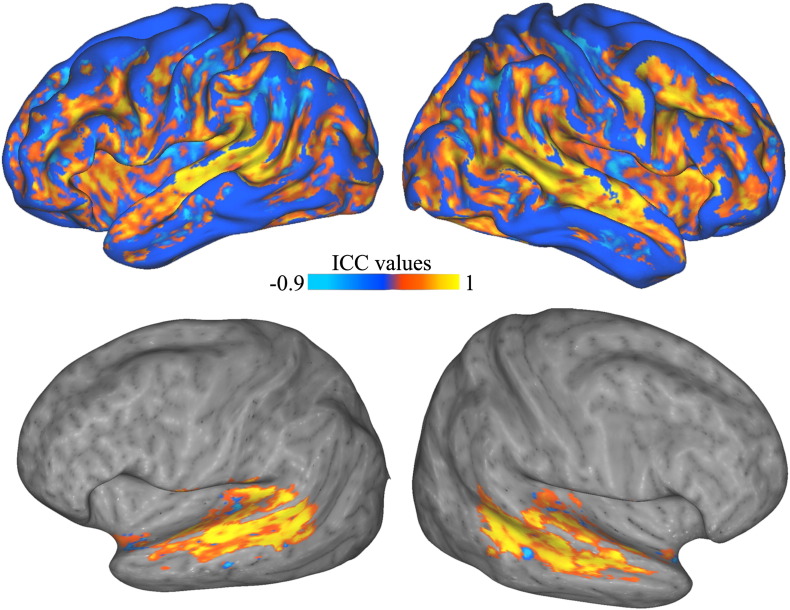
Test–Retest reliability. Intra-class correlation (ICC) coefficients obtained from the test–retest analysis of ten individual subjects projected onto the standard inflated cortical surface generated by Caret (Van Essen et al., 2001) and mapped in colormap without (top) and with thresholding (bottom). Note the very high reliability of voice-sensitive activity along the STS bilaterally.

ICC results in the ten subjects scanned twice show a good model consistency (positive ICC) over areas revealed by the random effect analysis, including STS/STG and inferior frontal gyri (Fig. 5). Highest ICC values (> 0.9) were obtained over the whole STS, suggesting high activation reliability of the TVA.

## Discussion

### A validated ‘voice localizer’ for individual localization of higher-level auditory cortical fields

We present and make available to the community a 10-min ‘voice localizer’ protocol for identification of voice-sensitive cortex in the human brain. Here we ‘validate’ this localizer showing that the TVA can be identified at the single-subject level in 94% of cases with a high test–retest correlation (> 0.9).

The experiment (stimuli and script), raw data and core analysis scripts at available on-line (http://dx.doi.org/10.7488/ds/280). Similalry the probabilistic and cluster maps of the TVA are available at: http://vnl.psy.gla.ac.uk/resources.php and all the results maps are on Neurovault (http://neurovault.org/collections/33/). The short duration of the voice localizer makes it suitable as an addition to specific auditory protocols as a means of individually identifying areas of higher-level auditory cortex in a standardized way: whereas procedures exist to identify core regions of auditory cortex based on the known tonotopic organization of these fields (Formisano et al., 2003, Moerel et al., 2012), no such procedure exists to date for identification of belt and para-belt areas of auditory cortex. The voice localizer aims to fill this gap and provide researchers with a standardized tool comparable to the localizers used to delineate higher-level fields of visual cortex (e.g., ‘face localizers’) (Kanwisher et al., 1997) allowing comparisons across sites and studies. Note that a shorter version of the localizer can also be used if time constraints are important, and still provides results of sufficient robustness particularly if Multi-Variate Pattern Analysis (MVPA) analysis is used (Ahrens et al., 2014).

There are many possible reasons why voices elicit stronger activations than non-voices, and the localizer scan is not intended to answer such questions which have been treated elsewhere (e.g., Andics et al., 2014, Belin et al., 2000). The voice localizer provides however a robust way to obtain significant activations at the individual level (Fig. 2), and these appear reliable as shown by test–retest analyses (Fig. 5). The localizer can be used for instance as a means of defining regions of interests independently of a main functional run, thereby avoiding circularity in statistical analyses (Kriegeskorte et al., 2009). The individual definition of ROIs can also be used as a means of increasing signal-to-noise ratio by reducing inter-subject variability (Pernet et al., 2007a). Note that the localizer is compatible both with a sparse sampling protocol using a TR of 10 s, and with continuous scanning protocol using a TR of 2 s.

### Limitations

There are several limitations to consider when using the voice localizer. First, from our design, subject passively listened to the stimuli and the order of stimuli was fixed. This leaves the door open to possible differences related to attention and arousal changes over time as well as carry over effects. Second, about 1/3 of vocal stimuli are speech – and these led to the strongest activations. This result implies that part of the sensitivity observed relates to speech processing. Indeed, we do not claim that the TVA are voice-specific, but that they show preferential responses to voice stimuli (Pernet et al., 2007b). In a meta-analysis comparing voice and speech, Samson et al. (2011) clearly showed an overlap of the temporal regions involved in both categories. It is conceivable that speech sensitivity is associated with the mechanisms that produce and perceive voices (McGettigan and Scott, 2012). Indeed, the ability to understand speech and the ability to recognize voices are often closely linked (Chandrasekaran et al., 2011, Perrachione et al., 2011). Finally, although the stimuli are matched in terms of energy (RMS), they can differ in many other acoustic factors such as amplitude modulation, interstimulus intervals, frequency bandwidth, and frequency modulation. Although some acoustical differences between vocal and non-vocal sounds had been ruled out as explanations for the observed differences (Belin et al., 2000), it remains possible that part of the difference observed relates to other acoustical factors. This does not however mean that the regions highlighted are not sensitive to voices, but that these regions process voice-specific acoustic features (Charest et al., 2013, Giordano et al., 2014, Latinus et al., 2013 for the most recent data).

### Three ‘voice patches’ along each temporal lobe

Single-subject analyses highlight significant clusters of greater BOLD signal in response to vocal compared to non-vocal sounds in the temporal lobes of 215 subjects out of 218, with considerable inter-individual variability (see e.g. Fig. 2). The probability map and a cluster analysis of the individual location of local vocal > non-vocal maxima confirms this large variability but highlights a clear pattern in the spatial organization: the peaks are not randomly distributed along the temporal cortex, but are organized in circumscribed clusters or ‘voice patches’ (Fig. 3, Fig. 4).

A broad region approximately in the middle of the right STS showed the strongest response on the RFX map (MNI: 60 − 14 0), a high cluster density (MNI: 53 − 18 3 TVAm), the maximum probability overlap (MNI 60 − 20/− 26 0: 85%), and also maximum ICC (> 0.9). This region is located below the planum temporale and caudally to Heschl's gyrus. Two other clusters, well distinct from this region were also observed more anterior (TVAa, max probability overlap 76%) and posterior (TVAp, max probability overlap 75%). Interestingly, TVAp showed the highest cluster density (but not maximum random effect *t*-value) and this location seems to correspond to the part of the STS where the depth is at its largest, providing a potentially interesting anatomical landmark. Further testing is however needed to confirm this hypothesis.

In the left hemisphere, the maximum of the RFX map (MNI: − 60 − 12 2) and of the probability map (MNI: − 60 − 14 0) point to a near symmetric region as the core right TVA, located in the middle of the STS. This region also shows maximum ICC (> 0.9). Contrary to the right hemisphere, it however appears that this region observed in volumetric analyses, is a mixture of responses coming from more anterior (TVAa – on the sulcus) and posterior (TVAm – on the gyrus) patches as revealed by the surface analysis. From the event-related analyses, no difference could be observed between the two regions in terms of response profile to different vocal and non-vocal categories. Differences were however observed when testing their selectivity to voices, suggesting only relative response homogeneity and caution should be taken when considering this region. Finally, like for the right hemisphere, a more posterior cluster was observed (TVAp, max probability overlap 60%). Overall, results suggest that the TVA are bilateral, largely symmetrical, with 3 patches per hemispheres and no lateralization effect. Although it is still not clear what is the exact role of these areas, current evidence suggests that the anterior and middle regions are more involved in voice-specific acoustical processing (e.g. Charest et al., 2013, Giordano et al., 2014, Latinus et al., 2013), and that posterior regions are more involved in audio-visual integration (Kreifelts et al., 2007, Kreifelts et al., 2009, Ethofer et al., 2013, Watson et al., 2014) but more work is needed to confirm this distinction.

### An extended voice processing network

The RFX analysis offers a fairly different picture to that suggested by individual TVA maps. While it only offers a blurred picture of the organization of voice patches in the temporal lobe, it highlights, thanks to the large sample size, a set or extra-temporal regions showing small, although significant, voice-sensitivity. Inspection of the anatomical location of these extra-temporal voice clusters, and their remarkable symmetry across the midline (Table 2) clearly suggests they are not trivial random effects. The RFX analysis in particular suggests the voice-sensitivity of a number of prefrontal regions, including a premotor region close to the frontal-eye fields and two distinct clusters of the inferior prefrontal cortex. All these areas are bilateral although comparisons of lowest vocal activity (emotional stimuli) vs. strongest non-vocal activity (music) showed stronger effects in the right hemisphere. Lateralization analyses showed however no hemispheric advantage at the group level with > 50% of subject having lateralization indices within the [− 0.2 0.2] interval. We expect this result to be true, no matter the participants' handedness (not recorded here). Voice processing, as language, is likely to be only weakly correlated with manual dominance (Mazoyer et al., 2014), and at best being partially pleiotropic (Ocklenburg et al., 2014). With only 10% of the population left handed and only 7–8% of them showing right hemispheric language dominance (Mazoyer et al., 2014), if the voice-selectivity and manual dominance were related to language, only 1 or 2 subjects should show right hemispheric dominance (vs. 20 to 30% observed). Similarly, assuming 75% of right handed subjects showing strong left language lateralization, we should have obtained 67% of left hemispheric lateralization (vs. 10 to 20% observed). If anything, the absence of lateralization reinforces the idea that voice information processing differs from language processing.

The observed frontal regions have been linked to perceived (rather than acoustic) voice distances (Charest et al., 2013) and seem to carry information about identity (Latinus et al., 2011), emotion (Ethofer et al., 2012, Frühholz and Grandjean, 2013a) and sound sources (Giordano et al., 2014). Interestingly, the IFG regions were also strongly interconnected with the TVAa, TVAm and the thalamus. Remarkably, this is the first time that voice sensitivity is observed in subcortical regions. The connectivity pattern of the thalamus suggests a modulation of the activity via both ascending and descending auditory pathways. Of interest here is the observed voice sensitivity of olivary nuclei, corroborating the report from Nan et al. (2015) showing talker sensitivity already present at this early processing stage of auditory information.

Remarkably, the amygdalae also show significant vocal vs. non-vocal response bilaterally. The interpretation of these voice-induced amygdala BOLD signal increases is not necessarily best formulated in terms of affective processing: although the amygdalae have been implicated in processing of threat-related stimuli such as angry or fearful faces or voices (Frühholz and Grandjean, 2013b, Morris et al., 1996, Scott et al., 1997) it is increasingly recognized that other emotions, including positive ones, also engage the amygdala (e.g. Fecteau et al., 2007) and that the function of the amygdala could be more accurately described as a ‘relevance detector’ (Sander et al., 2003). This is in line with our results which show that amygdalae are sensitive to voices, and not necessarily to affectively loaded stimuli, but instead reflect the extremely high importance of the human voice as a stimulus category in our social environment. In addition, connectivity analyses show that the strongest connections to the amygdala were directly from the thalamus (significant for the left amygdalae and under threshold for the right amygdala) but not from the TVA. Interestingly a similar result has been observed for presentation of neutral faces, which do not typically activate the amygdala when they do not express any emotion, yet have been found to reliably engage an extended ‘face network’ including the amygdalae when analyzed in a large cohort (Todorov and Engell, 2008).

The following are the supplementary data related to this article.

Supplementary Figure 1 Whole brain pair-wise comparisons between vocal and non-vocal sub-categories (p<=0.05 FWE corrected).

Supplementary Tables 1 and 2 Mean Percentage signal change and bootstrapped 95% confidence intervals estimated for each category of vocal and non-vocal stimuli (scaling factor = 0.0132).

## Figures and Tables

**Table 1:** Stimuli characteristics. The amplitude range corresponds to the distance between the lowest and highest peaks in the time domain. The frequency peak indicates where the maximum energy was located in the frequency spectrum.

	Durations (s)	Nb of stimuli	Amplitude range (dB)	Frequency peak (Hz)
Vocal sounds	8 s blocks	1 blocks of 3 5 blocks of 4 10 blocks of 5 4 blocks of 6	− 5 [− 12 5]	660 [142 3275]
Emotionally neutral	1.8 s [0.2 2.5]	39	− 5 [− 12 5.7]	659 [142 2619]
Emotionally loaded	2 s [1.1 3.5]	27	− 3 [− 8.8 5]	859 [178 3275]
Speech	0.65 s [0.2 4]	31	− 7 [− 10 -3.8]	464 [178 886]
*Non-vocal*	*8* s *blocks*	*2 blocks of 4* *17 blocks of 5* *1 block of 6*	*− 5* *[− 15 4]*	*928.5* *[60 7916]*
Animals	1.5 s [1.1 2.5]	29	− 6 [− 12 -1]	1368 [161 4723]
Natural	1.75 s [1.2 2.6]	18	− 5 [− 11 1]	397 [60 1676]
Man-made	1.4 s [0.4 2.6]	41	− 6 [− 15 4]	1528 [60 7919]
Music	1.55 s [0.8 2.5]	10	− 6 [− 11 -3]	421 [85 892]

**Table 2:** RFX results. The table shows (i) MNI coordinates of local maxima (peaks separated by more than 8 mm, 3 peaks max listed per cluster), (ii) the corresponding *t*-value of the vocal > non-vocal contrast at that location (height threshold *t*(1, 217) = 4.79 *p* < 0.05 FWE corrected), (iii) the cluster size, (iv) the anatomical regions within the clusters, and (v) percentage signal change with bootstrap 95% confidence intervals for each ROI (TVA anterior, mid, and posterior (33 voxel each), the IFG ventral (left: 231 voxels, right: 71 voxels), and medial (left: 515 voxels, right: 1039 voxels), the precentral gyrus (left: 106 voxels, right: 393 voxels), the thalamus (left: 108 voxels, right: 227 voxels), amygdalae (left: 96 voxels, right: 95 voxels) and olivary nuclei (left: 67 voxels, right: 92 voxels)).

*x y z*	*t*	Cluster size	Labeling	Mean PSC difference and 95% CI
− 60 − 12 2	25.06	4256	Left superior temporal Gyrus and sulcus (TE3,TE1) Left amygdala	TVAa 0.0039 [0.0036 0.0043] TVAm 0.004 [0.003 0.0043] TVAp 0.0017 [0.0015 0.0019] 0.001 [0.0008 0.0013]
− 62 − 22 4	23.64
− 56 2 − 10	18.80
60 − 14 0	25.24	4802	Right superior temporal Gyrus and sulcus Right inferior frontal ventral Right inferior frontal medial Right precentral	TVAa 0.0035 [0.0031 0.0038] TVAm 0.0039 [0.0037 0.0042] TVAp 0.0021 [0.002 0.0024] 0.0009 [0.0007 0.001] 0.0014 [0.0012 0.0017] 0.0013 [0.0011 0.0016]
60 − 26 0	24.08
60 0 − 4	21.24
− 50 − 6 46	11.30	108	Left precentral gyrus	0.0011 [0.0009 0.0014]
− 40 28 − 2	10	233	Left inferior frontal gyrus ventral	0.0011 [0.0008 0.0014]
20 − 8 − 12	9.85	84	Right amygdala	0.0009 [0.0007 0.001]
26 0 − 18	5.73
− 46 14 24	9.60	500	Left inferior frontal gyrus medial	0.0013 [0.001 0.0016]
12 − 14 8	8.50	419	Left thalamus Right thalamus	0.00072 [0.0005 0.0009] 0.00079 [0.0005 0.001]
14 − 4 10	6.56
8 − 4 2	6.48
14 − 26 − 6	8.20	92	Right pons (olivary nucleus)	0.001 [0.0007 0.0013]
6 − 32 0	5.53
− 14 − 26 − 6	6.82	65	Left pons (olivary nucleus)	0.0008 [0.0005 0.001]

**Table 3:** MNI coordinates of TVA peak clusters or ‘voice patches’ observed in the cluster analysis (Fig. 4).

*x y z*	Voice patch	Labeling
42 − 35 3	Right TVAp	Right middle/ posterior superior temporal gyrus
53 − 18 − 3	Right TVAm	Middle superior temporal sulcus/gyrus
55 − 2 − 7	Right TVAa	Anterior superior temporal sulcus
− 46 − 38 2	Leftg TVAp	Middle/posterior superior temporal gyrus
− 55 − 18 − 3	Left TVAm	Middle superior temporal gyrus
− 55 − 8 − 3	Left TVAa	Anterior superior temporal sulcus
