# Supplementary material for: The human voice areas: Spatial organization and inter-individual variability in temporal and extra-temporal cortices
Source: Neuroimage. 2015 Oct 1;119:164–74. doi: 10.1016/j.neuroimage.2015.06.050 (PMC4768083; doi:10.1016/j.neuroimage.2015.06.050)
Supplement: Supplementary Tables 1 and 2 — Mean Percentage signal change and bootstrapped 95% confidence intervals estimated for each category of vocal and non-vocal stimuli (scaling factor = 0.0132). [file mmc2.docx]

|  | **Emotional** | **Neutral** | **Speech** |
| --- | --- | --- | --- |
| Left IFG ventral | 0.0006  [0.0002 0.0011] | 0.0014  [0.0010 0.0017] | 0.0026  [0.0019 0.0033] |
| Right IFG ventral | 0.0010  [0.0005 0.0016] | 0.0012  [0.0008 0.0016] | 0.0020  [0.0014 0.0026] |
| Left IFG medial | 0.0012  [0.0007 0.0017] | 0.0021  [0.0017 0.0025] | 0.0041  [0.0034 0.0049] |
| Right IFG medial | 0.0020  [0.0015 0.0025] | 0.0027  [0.0023 0.0031] | 0.0043  [0.0037 0.0050] |
| Left precentral | -0.0002  [-0.0007 0.0003] | 0.0015  [0.0010 0.0020] | 0.0029  [0.0021 0.0037] |
| Right precentral | 0.0015  [0.0010 0.0020] | 0.0025  [0.0020 0.0029] | 0.0031  [0.0025 0.0036] |
| Left TVAa | 0.0061  [0.0054 0.0069] | 0.0109  [0.0101 0.0117] | 0.0151  [0.0141 0.0161] |
| Right TVAa | 0.0055  [0.0049 0.0061] | 0.0089  [0.0083 0.0096] | 0.0126  [0.0116 0.0136] |
| Left TVAm | 0.0045  [0.0039 0.0051] | 0.0077  [0.0071 0.0083] | 0.0121  [0.0111 0.0131] |
| Right TVAm | 0.0065  [0.0057 0.0073] | 0.0100  [0.0091 0.0108] | 0.0137  [0.0127 0.0148] |
| Left TVAp | 0.0025  [0.0019 0.0031] | 0.0049  [0.0043 0.0054] | 0.0082  [0.0073 0.0090] |
| Right TVAp | 0.0051  [0.0043 0.0060] | 0.0077  [0.0068 0.0087] | 0.0106  [0.0104 0.0129] |
| Left amygdala | 0.0016  [0.0011 0.0021] | 0.0019  [0.0014 0.0023] | 0.0022  [0.0017 0.0028] |
| Right amygdala | 0.0014  [0.0010 0.0019] | 0.0017  [0.0013 0.0022] | 0.0021  [0.0016 0.0027] |
| Left olive | 0.0019  [0.0014 0.0023] | 0.0028  [0.0024 0.0033] | 0.0032  [0.0026 0.0038] |
| Right olive | 0.0027  [0.0021 0.0032] | 0.0036  [0.0031 0.0041] | 0.0037  [0.0030 0.0044] |
| Left thalamus | 0.0011  [0.0007 0.0015] | 0.0014  [0.0010 0.0018] | 0.0012  [0.0007 0.0017] |
| Right thalamus | 0.0013  [0.0010 0.0017] | 0.0015  [0.0012 0.0019] | 0.0014  [0.0010 0.0019] |

Supplementary Table 1. Mean Percentage signal change and bootstrapped 95% confidence intervals estimated for each category of vocal stimuli (scaling factor = 0.0132).

|  | **Animal calls** | **Man-made object sounds** | **Music** | **Natural sounds** |
| --- | --- | --- | --- | --- |
| Left IFG ventral | 0.0004  [-0.0001 0.0008] | -0.0002  [-0.0005 0.0002] | 0.0015  [0.0008 0.0022] | -0.00004  [-0.0005 0.0005] |
| Right IFG ventral | 0.0008  [0.0004 0.0013] | -0.0001  [-0.0005 0.0002] | 0.0014  [0.0006 0.0021] | 0.000002  [-0.0005 0.0005] |
| Left IFG medial | 0.0012  [0.0007 0.0016] | 0.0004  [-0.0003 0.0008] | 0.0024  [0.0016 0.0032] | 0.0003  [-0.0003 0.0009] |
| Right IFG medial | 0.0018  [0.0014 0.0022] | 0.0007  [0.0003 0.0011] | 0.0035  [0.0028 0.0041] | 0.0004  [-0.0001 0.0009] |
| Left precentral | 0.0002  [-0.0003 0.0006] | -0.0005  [-0.0010 0.0010] | 0.0009  [0.00001 0.0017] | 0.0001  [-0.0004 0.0006] |
| Right precentral | 0.0011  [0.0007 0.0015] | 0.0003  [0.0001 0.0007] | 0.0026  [0.0019 0.0033] | 0.0005  [0.00001 0.0010] |
| Left TVAa | 0.0080  [0.0072 0.0087] | 0.0055  [0.0048 0.0062] | 0.0103  [0.0094 0.0113] | 0.0030  [0.0024 0.0037] |
| Right TVAa | 0.0064  [0.0058 0.0070] | 0.0043  [0.0038 0.0048] | 0.0089  [0.0080 0.0097] | 0.0019  [0.0014 0.0024] |
| Left TVAm | 0.0048  [0.0043 0.0054] | 0.0023  [0.0018 0.0028] | 0.0053  [0.0045 0.0060] | 0.0017  [0.0012 0.0022] |
| Right TVAm | 0.0056  [0.0050 0.0063] | 0.0029  [0.0023.0036] | 0.0072  [0.0061 0.083] | 0.0013  [0.0007 0.0019] |
| Left TVAp | 0.0026  [0.0021 0.0031] | 0.0013  [0.0008 0.0018] | 0.0031  [0.0024 0.00382] | 0.0011  [0.0007 0.0016] |
| Right TVAp | 0.0031  [0.0023 0.0038] | 0.0014  [0.0008 0.0020] | 0.0059  [0.0048 0.0070] | 0.0003  [-0.0003 0.0009] |
| Left amygdala | 0.0013  [0.0007 0.0018] | 0.0003  [-0.0002 0.0007] | 0.0020  [0.0011 0.0029] | -0.0001  [-0.0008 0.0006] |
| Right amygdala | 0.0013  [0.0009 0.0018] | 0.0003  [-0.0002 0.0006] | 0.0018  [0.0010 0.0027] | 0.00002  [-0.0005 0.0006] |
| Left olive | 0.0030  [0.0024 0.0035] | 0.0008  [0.0003 0.0013] | 0.0019  [0.0010 0.0028] | 0.0011  [0.0005 0.0018] |
| Right olive | 0.0038  [0.0032 0.0044] | 0.0014  [0.0008 0.0020] | 0.0028  [0.0019 0.0038] | 0.0012  [0.0005 0.0020] |
| Left thalamus | 0.0013  [0.0009 0.0018] | -0.0001  [-0.0005 0.0003] | 0.0007  [-0.0001 0.0014] | 0.0013  [-0.0002 0.0009] |
| Right thalamus | 0.0015  [0.0010 0.0019] | 0.000001  [-0.0004 0.0003] | 0.0012  [0.0004 0.0019] | 0.0004  [-0.0002 0.0009] |

Supplementary Table 2. Mean Percentage signal change and bootstrapped 95% confidence intervals estimated for each category of non-vocal stimuli (scaling factor = 0.0132).
